# Non-Pharmacological Treatment of Cardiovascular Disease | Importance of Physical Exercise

**DOI:** 10.5935/abc.20190118

**Published:** 2019-07

**Authors:** Mariana Janini Gomes, Luana Urbano Pagan, Marina Politi Okoshi

**Affiliations:** Faculdade de Medicina de Botucatu, Universidade Estadual Paulista (UNESP), Botucatu, SP - Brazil

**Keywords:** Cardiovascular Diseases, Heart Failure, Exercise/prevention and control, Physical Fitness, Ventricular Remodeling, Quality of Life

Cardiovascular diseases are currently the major health problem and are directly involved in more than 17 million deaths each year, which represents 50% of all deaths from noncommunicable diseases.^1^ In addition to their effects on individual well-being, cardiovascular diseases are responsible for a high economic impact. A recently published study showed that in Brazil, only four diseases - arterial hypertension, myocardial infarction, atrial fibrillation and heart failure - reached an estimated total financial cost of 56.2 billion reais in the year 2015.^1^

The treatment of cardiovascular diseases involves the use of specific drugs and adherence to non-pharmacological interventions.^2^ This Editorial will be dedicated to the role of physical exercise in the treatment of cardiovascular diseases.

Brazil has a prominent position worldwide regarding the study of the effects of physical exercise in different clinical conditions. The practice of physical exercises has been recommended for decades for health promotion and treatment of several cardiovascular diseases. The regular practice of exercises results in several benefits, such as increased functional capacity and improved body composition, insulin resistance, endothelial function, arterial hypertension, antioxidant status, and quality of life.^3-9^

Concerning heart failure, exercises have been recommended for almost three decades in the treatment of stable patients. In addition to increasing effort tolerance, it improves the quality of life and reduces hospitalizations for heart failure.^2^ Despite a large number of studies evaluating the effects of exercise, its influence on different situations of cardiac aggression has yet to be fully clarified.^10^

In articles recently published in this journal, the role of exercise and its molecular mechanisms of action have been evaluated in different heart disease experimental models.^3,5,11-13^ The beneficial effects on cardiac remodeling have been frequently observed, such as attenuation of myocardial hypertrophy and left ventricular dysfunction.^7,14^ However, unexpected results have drawn attention to the need for better clarification of the subject. For instance, Rodrigues et al.^13^ submitted beta-adrenergic receptor knockout mice, which may develop heart failure, to treadmill training for eight weeks. Surprisingly, the trained knockout animals showed a higher increase in functional capacity and myocardial contractility than the trained control animals. As an exaggerated contractility stimulus may lead to deterioration of cardiac function in the long-term, additional studies are required to define the role of exercise in cardiac function in beta-adrenergic receptor knockout mice in later life.

Topics of great uncertainty regarding exercise prescription include the intensity and duration of exercise. Recently, Ellingsen et al.^15^ published the first multicenter randomized trial comparing the effects of high-intensity interval training (HIIT) with those of continuous training at moderate intensity or recommendation for regular exercises in patients with heart failure with reduced ejection fraction. In both specific training groups, the results were only moderately better than the recommendation for regular exercise. Moreover, 51% of patients in the HIIT group exercised below the prescribed heart rate, and 80% of the individuals from continuous training group at moderate intensity trained at a frequency above their target. Thus, considering that HIIT was not superior to the continuous training group at moderate intensity in reducing the remodeling process or improving clinical outcomes, and the difficulty in attaining adherence to the prescribed intensity, the authors recommend that continuous training at moderate intensity should remain as the standard modality for patients with chronic heart failure.

Another factor that remains to be clarified is whether the practice of exercise in short and intense periods repeated throughout the day, called accumulated exercise, can be an alternative for sedentary individuals. Martinez et al.^16^ observed that both continuous and accumulated exercise improved the physical fitness of healthy rats. However, only the continuous exercise was able to reduce body weight gain and improve endothelial function. Aortas obtained from the group submitted to continuous exercise showed a reduction in the contractile response to norepinephrine and an increase in acetylcholine-induced relaxation, which was not observed in the group trained using accumulated exercise.^16^

A greater consensus is observed in the literature regarding the role of physical exercise on the vascular system. Lemos et al.^12^ showed that regular aerobic exercise for nine weeks led to the attenuation of sympathetic activity and reduction in vascular resistance, thus contributing to a decrease in blood pressure in spontaneously hypertensive rats. Resistance training was also effective in improving the bradycardic response and baroreflex sensitivity of spontaneously hypertensive rats.^11^ However, the fact that these effects were not accompanied by a reduction in systemic blood pressure ^11^ suggests that aerobic exercise is superior to resistance training for arterial hypertension control.

Despite the great advances regarding the understanding of physical exercise effects on the healthy cardiovascular system or that submitted to different types of aggression, we are still far from clarifying the physical exercise mechanisms of action and from scientifically defining the best prescription for patients with cardiovascular disease.
